# Temporal Structure of Human Gaze Dynamics Is Invariant During Free Viewing

**DOI:** 10.1371/journal.pone.0139379

**Published:** 2015-09-30

**Authors:** Colleen A. Marlow, Indre V. Viskontas, Alisa Matlin, Cooper Boydston, Adam Boxer, Richard P. Taylor

**Affiliations:** 1 Keck Center for Integrative Neuroscience, University of California, San Francisco, California, 94143–044, United States of America; 2 Physics Department, California Polytechnic State University, San Luis Obispo, California, 93407, United States of America; 3 Memory and Aging Center, University of California, San Francisco, California, 94143–044, United States of America; 4 Physics Department, University of Oregon, Eugene, Oregon, 97403–1274, United States of America; Universitat de Valencia, SPAIN

## Abstract

We investigate the dynamic structure of human gaze and present an experimental study of the frequency components of the change in gaze position over time during free viewing of computer-generated fractal images. We show that changes in gaze position are scale-invariant in time with statistical properties that are characteristic of a random walk process. We quantify and track changes in the temporal structure using a well-defined scaling parameter called the Hurst exponent, *H*. We find *H* is robust regardless of the spatial complexity generated by the fractal images. In addition, we find the Hurst exponent is invariant across all participants, including those with distinct changes to higher order visual processes due to neural degeneration. The value we find for *H* of 0.57 shows that the gaze dynamics during free viewing of fractal images are consistent with a random walk process with persistent movements. Our research suggests the human visual system may have a common strategy that drives the dynamics of human gaze during exploration.

## Introduction

The fovea is the region of the retina with the highest visual acuity. To allow the human neural system to sample its visual environment, the gaze, or line of sight from the fovea to the position of interest in the visual field, is adjusted over time. The movements of the gaze within the visual field form a trajectory, known as the scan path, which is composed of a series of rapid changes in gaze position. Eye tracking technology can measure the position of the gaze in the visual field, allowing participants’ scan paths to be tracked while they engage in visual tasks in real-time. Using this technology, the human eye’s scan path is observed to have a complex structure both spatially and temporally. The underlying mechanisms in the neural system which modulate how the eye navigates the visual field to generate this complex structure is a topic of much discussion [1]. The structure of human gaze changes is thought to arise from the interplay of bottom-up factors such as the saliency of image features and top-down higher order cognitive factors such as those affecting attention. Historically, models tend to focus on the role of visual attention to predict where localizations of the gaze, fixations, occur within a visual scene [2]. However, in order to understand the complex dynamics of the human visual system as it navigates the sensory field it is necessary also to take into account how the gaze shifts occur in time. Recent models take into account the importance of the temporal dynamics of gaze by modelling gaze shifts using random walk processes [3–5].

A random walk involves a series of steps for which the root mean square displacement,〈*d* ^2^〉, of the walker scales with the elapsed time, *t*, following the relationship 〈*d* ^2^〉~*t* ^2*H*^. The specific value of the Hurst exponent *H* determines how the random walk evolves in time and is related to the probability distribution of a random walk process. It determines how the trajectory of the walk fills space over time and the diffusive properties of the process. For example, a value of *H* = 1/2 is the signature of a Gaussian distribution yielding Brownian motion with normal diffusion. In contrast, values of *H* > 1/2 are linked to long-tailed distributions (i.e. Lévy Flights [6]) with superdiffusive behavior and values of *H* < 1/2 are associated with subdiffusive behavior.

Models of gaze shifts based on random-walk processes predict scale invariance in gaze dynamics. Recent experiments confirmed scale invariance in the temporal structure of the larger shifts in gaze position [6–9], referred to as saccades. Additionally, scale invariance has also been observed in so-called fixational eye movements [10, 11] which are the microscopic shifts in gaze that occur while the eye is fixating on a localized region in the visual field. Recent models incorporating the prevalent feature of temporal scale invariance show promise in uncovering the underlying dynamics employed by the visual system during fixation [12] and have provided a better understanding of the function of fixational movements in seeing [13].

The success of the temporal analysis of gaze shifts during fixation motivates our study on the temporal qualities of changes in gaze which occur during free viewing. We apply a time-series analysis method that reliably identifies and quantifies any changes in the temporal scale invariance of gaze shifts. This allows us to describe, characterize and compare the temporal properties of the movements across individual participants engaged in visual exploration during free viewing of computer-generated fractal images.

The prevalence of fractals in our natural environment has motivated a number of investigations of human behavioral and neurophysiological responses to their visual characteristics. Fractals feature patterns that repeat at increasing fine size scales, building immense visual complexity. Examples of these scale invariant patterns include tree branches, coastlines, clouds, and mountain profiles. In our study, we use computer-generated fractal images to establish the basic temporal gaze structure during free viewing and then present preliminary data indicating that this behavior extends to the more subtle stimuli of natural scenes.

This particular viewing scenario was chosen such that higher order processes are not strongly engaged during visual exploration. Low level pre-attentive image features dominate gaze control during free viewing of static images [14, 15]. Within this context, we vary the spatial complexity of the salient feature of our fractal images- which are the fractal edges of the black and white regions.

We identify scale invariance of the gaze shifts and determine the Hurst exponent associated with the time series using the variation method, as described in the Analysis section. We then track the Hurst exponents to determine whether the overall strategy of the scan path depends on the spatial complexity of the fractal image or solely on the viewing mode the individual is engaged in. Participants with neural degenerative disease were included to assess whether physical changes in the visual system due to the disease affect the overall gaze dynamics. By identifying when and if the temporal dynamics of the scan path change with neural degeneration will help determine if the gaze shift strategy is controlled for at a higher level in the visual system or at the oculomotor level. We will contrast our results, in which participants were not intentionally fixating, with studies in which participants were actively fixating. Then we discuss this contrast in the context of the function of the visual system.

## Materials and Methods

### Participants

Gaze position data was collected for 20 participants, 11 of whom were healthy participants (H) and 9 were participants with neural degenerative (ND) diseases. ND participants comprised 3 participants with Alzheimer’s disease (AD), 5 participants with degeneration in the frontal and anterior temporal lobes (FTD) and 1 participant with progressive supranuclear palsy (PSP). Of the H group, 8 were young normal (YN) participants (ages ranging from 18 to 35) and 3 were age-matched normal control (NC) participants for comparison to ND patients. Participants were recruited and evaluated through the Memory and Aging Center at University of California, San Francisco (UCSF). Neurodegenerative participants were diagnosed by a team of neurologists, neurophysiologists and nurses after extensive examination. Participants with red/green colorblindness or who had undergone eye surgery were excluded from the study.

All participants involved in this research gave informed written consent to participate in the experimental procedures. In cases where participants had a compromised capacity to provide consent, a legally authorized representative gave informed written consent on behalf of the participant. This study was approved by the University of California, San Francisco Institutional Review Board.

### Experiment Procedure

**Fig 1:**
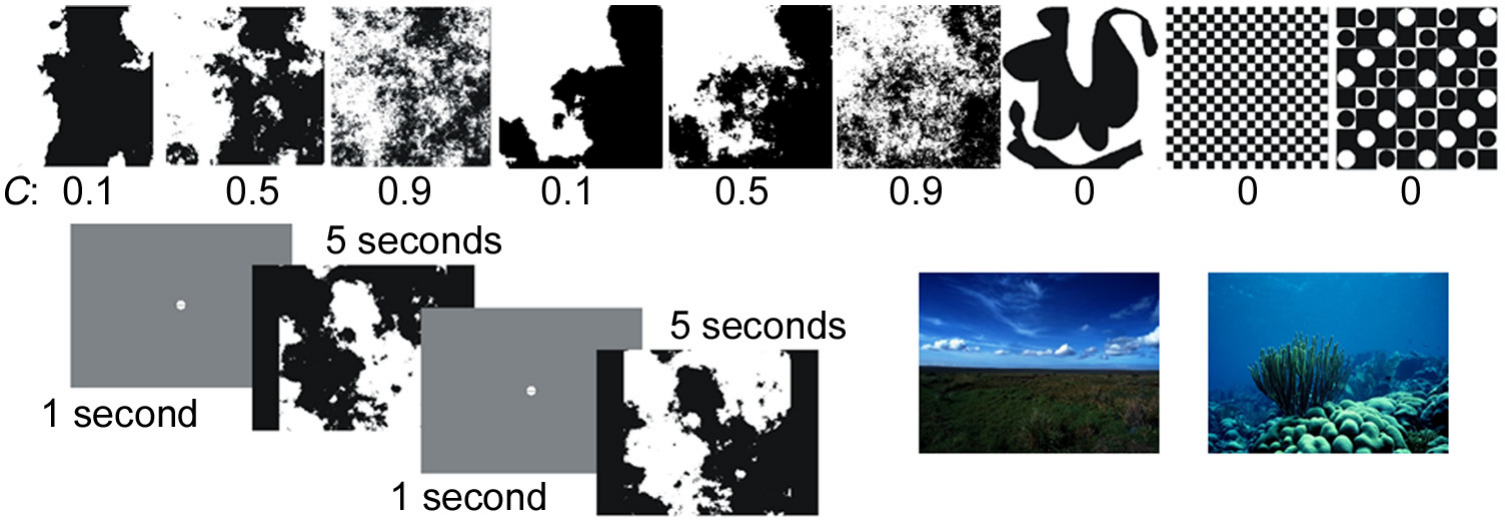
Examples of different fractal image stimuli which vary in spatial complexity from low (*C* = 0.1) to high (*C* = 0.9), along with examples of non-fractal images used (*C* = 0). Also shown in the lower left is the image viewing protocol, where participants focus on the screen center for one second between images. The lower right has two examples of natural landscape images used.

Each participant viewed from 9 to 93 images including at least one complete set of fractal images of varying complexity (Fig 1). The images, along with versions of the image where the black and white regions were inverted, were presented in random order. Each image was viewed for 5 seconds and a fixation screen was viewed for 1 second between each image (to re-center the gaze). The viewing protocol is shown in the lower left section of Fig 1. To increase the likelihood the participant remained engaged throughout the entire viewing time, participants were asked to answer (recorded with a press of key on a keyboard) whether or not they found the image aesthetically appealing as compared to previous images. The x and y positions of the participant’s left pupil’s gaze were recorded at 120 Hz using an ASL infrared eye tracker (504HS).

It is important to note that gaze position data collected in this study is therefore limited to changes on times scales of 8.3 ms and above. As a result, we do not probe the entire temporal range in which fixational movements extend, typically down to 2ms [10–12]. However, we are still able to observe shifts over a significant range of measured timescales, from 8.3ms to 5s.

Images were displayed on a LCD monitor (30.48 cm by 40.64 cm) which participants viewed at a distance of 67.3 cm. A chin rest and head strap were used to keep the participant’s head steady and the screen to pupil distance fixed throughout viewing. All images were 768 x 768 pixels subtending the visual angles of 24° vertically and 24° horizontally (one image pixel subtended an angel of 0.034° in the visual field). The upper value of the noise in the measured gaze position was 0.03°, just under the pixel resolution.

Using our computer-generated fractal images we can control the spatial complexity easily by altering the fractal dimension, *D*, of the image. We speculate that images with a large range of spatial complexity will induce changes in the gaze dynamics responsible for exploration. In particular, we hypothesize that as image complexity is increased the Hurst exponent will increase to accommodate. Images with higher *D* have higher ratios of fine to coarse spatial structure which in turn generates a higher visual spatial complexity. Fig 1 shows examples of typical fractal images used (top row) along with Euclidean (non-fractal) example images. We quantify the complexity, *C*, of the fractal images as *C* = *D– 1*. A non-fractal image has a value of *D* = 1 and therefore a low complexity of *C* = 0. A fractal image with *D* = 1.9 has a high complexity of *C* = 0.9. Images ranged from *C* = 0 to 0.9 in increments of 0.1. The non-fractal images were either simple geometric shapes, abstract images or patterns of geometric shapes (see Fig 1 top left for examples).

### Analysis

All raw gaze position data files were processed after collection to eliminate files with either multiple or long events where the gaze was lost by the eye tracker and cases for which the raw gaze position exceeded the image boundaries (i.e. the gaze position was off-screen) for over 110 ms (the average length of a blink). The magnitude of the change in gaze position with time, *l*, is calculated from the vertical and horizontal coordinates of the gaze position of two successive points in time (*x*_*i+1*_, *y*_*i+1*_ and *x*_*i*_, *y*_*i*_) and is defined as $l\;=\;\sqrt{{\left(x_{i+1}-x_{i}\right)}^{2}+{\left(y_{i+1}-y_{i}\right)}^{2}}$.

#### Variation Method

We use the variation method to identify whether the gaze data exhibits the scale invariance characteristic of random walk processes. The variation method is a well-established spectral analysis method appropriate for time-series data [16]. It not only reliably detects scale invariance but will allow us to quantify the Hurst exponent associated with the random walk process and detect any changes to this value. In the variation method, the data trace is covered with columns of spacing Δ*t* and then the minimum number of non-overlapping boxes, *N*, needed to cover the data structure is calculated. This process is repeated for increasingly fine time scale until the data resolution limit is reached. If the time series is scale invariant then *N*(Δ*t*) follows a power law of the form

$$N(\Delta t)\sim {\Delta t}^{-(2-H)}$$

over a significant range of timescales, at least one order of magnitude. Scale invariance and the associated Hurst exponent *H* value can be determined from a plot of-*log N*(Δ*t*) vs. *log* (Δ*t*). A linear plot demonstrates scale invariance and *H* is determined from the slope of the line. Shallower slopes (*H* closer to 1) indicate a bias toward low frequency movements and steeper slopes (*H* closer to 0) indicate a bias toward high frequency movements.

The variation method differs from basic box counting methods [17] because the grid height is normalized to account for the different scaling factors implicit in each of the coordinates defining the structure (i.e. the variable *l* does not have the same units as *t*). This important difference is what makes it possible for the variation method to assess the scale-invariant behavior of the times series. The variation method is also optimized to avoid over-counting of the structure while maximizing counting statistics leading to a more accurate value of the scaling exponent. Our approach is similar in concept to determining the power spectrum where one looks at the frequency content of time series. Using power spectrum analysis, scale invariance occurs when a data trace’s power is an inverse function of frequency, S(*f*)~1/*f* ^*β*^. For a finite set of data points statistics are limited. In this case, the power spectrum analysis leads to greater scatter in the output function when compared to the variation method, rendering the variation method the more accurate of the two methods [16]. A computational algorithm was developed and performed in MATLAB to apply the variation method analysis to the change in gaze position data with time, *l*(*t*), collected in this study.

## Results

**Fig 2:**
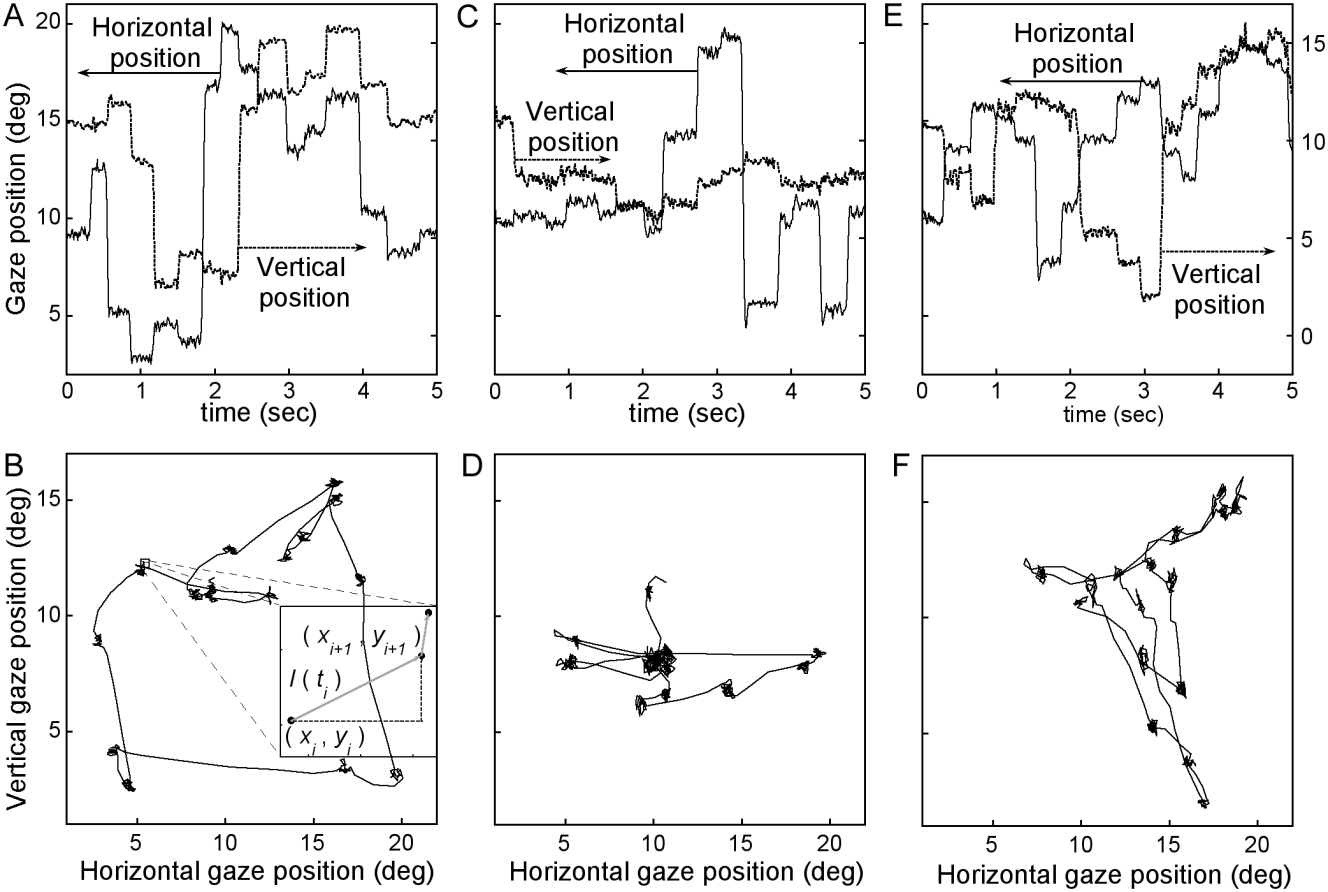
Sample gaze position and scan path data and plots of the scan paths of the same data below. Vertical and horizontal gaze position recorded for the five seconds of viewing for participant (A) Y1 and (C) FTD5 viewing a computer-generated fractal image and (E) FTD5 viewing a natural landscape. Along with plots of the scan path for (B) Y1, (D) FTD5 and (E) FTD5 of the accompanying data. The inset in (B) illustrates the magnitude of change in gaze position, *l*, between two gaze position data points in time.

**Fig 3:**
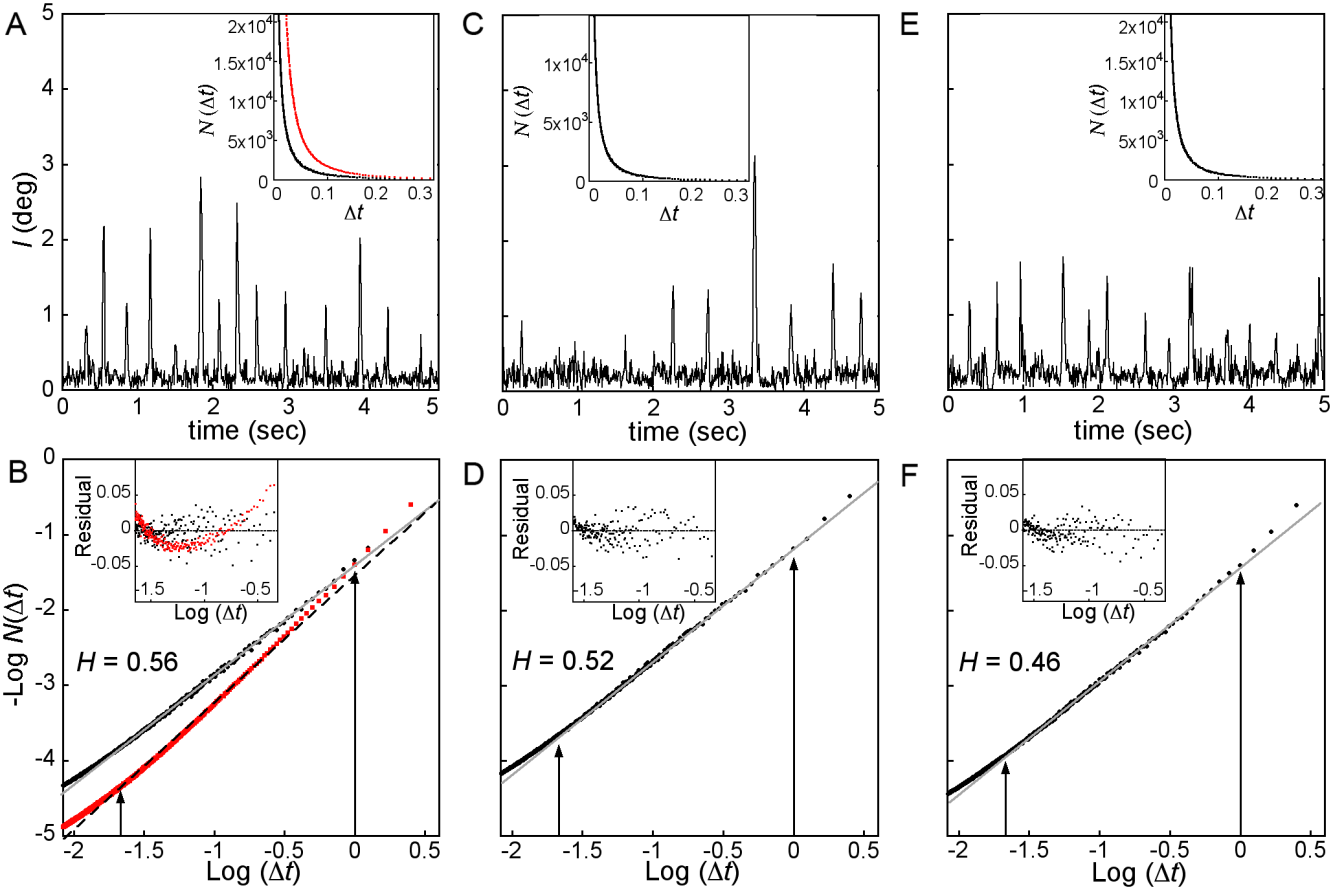
Calculated change in gaze position, *l*, as a function of time along with resulting variation method plot of *N*(Δ*t*) in inset for (A) participant Y1, (C) FTD5 viewing the computer-generated fractal image and (E) FTD5 viewing the natural landscape. Actual data is shown as black circles and surrogate data in red squares. Note time axis in insets have been cropped in order to see details of plot. The associated plot of–log *N*(Δ*t*) vs. log Δ*t* for (B) the actual and surrogate data for participant Y1, and the data for FTD5, (D) and (F). The linear behavior observed in the actual data demonstrates the scale invariance of *l (t)* within the measured range. The Hurst exponent *H* is listed on the plots and was obtained from the slope of the linear fit (slope = 2-*H*). Insets shows residuals of the linear fits.

Data from the healthy participant Y1 is used as a typical example of observations. Fig 2A and 2B show the participant’s vertical and horizontal gaze position along with the accompanying scan path. The magnitude of the change in gaze position with time, *l(t)*, for this data is shown in Fig 3A. The variation method output *N*(Δ*t*) is plotted as a function of Δ*t*, and follows an inverse power law. This can be clearly observed in the linearity of the plot of–log *N*(Δ*t*) with log Δ*t* (black circles in Fig 3B). The data is plotted within the observational time limits of the experiment and the linear fit to the data is shown as a solid line. The scaling exponent for this particular gaze data is found to be 1.44 by a linear regressive fit of the scaling plot, giving a Hurst exponent value of *H* = 0.56. The plot is linear over the range marked by the solid arrows indicating *l* (t) is scale invariant across 1.5 orders of magnitude, consistent with observations of scale invariance in other physical systems in nature [18]. The observational time cut-offs are set by the length of time participants viewed the image (5 seconds) and the time resolution (8.3 ms) of the data collection. The deviations we see in the data from the linear, scale invariant behavior occur at the positions along the x-axis, marked by the arrows, where we would expect them to be based on the statistical criteria of our analysis. In order to reliably detect scale invariant structure we need at least a grid size of 1/5 of the maximum trace length (5 seconds). The coarse-scale observational cut-off in our study is thus 1 s, as marked by the arrow on Fig 3B. We also cannot detect structure below times which are 3 times the resolution (8.3 ms) of the data collected. This fine-scale cut-off is also marked on Fig 3B.

To ensure that the observed scale invariance is not an artifact of the measurement system we created surrogate data by randomly shuffling the actual gaze position data in time. The scaling plot associated with the surrogate data is plotted as red squares in Fig 3B (bottom plot). The failure of the surrogate data to follow a power law across the observable range is best illustrated by the plot of the residuals in the inset of Fig 3B. The plot shows a U-shape consistent with a nonlinear log-log plot. This demonstrates that the scale invariance is removed through random shuffling and is therefore not an artifact of the measurement system but rather a signature of human gaze dynamics. This temporal scale invariance across participants is consistent with previous results [7–10, 17]. Equivalent plots were generated for all gaze position data collected and linearity was seen for all images and for all participants. Fig 2C and 2D and Fig 3C and 3D show the equivalent plots for participant FTD5 while also looking at a computer-generated fractal.

**Fig 4:**
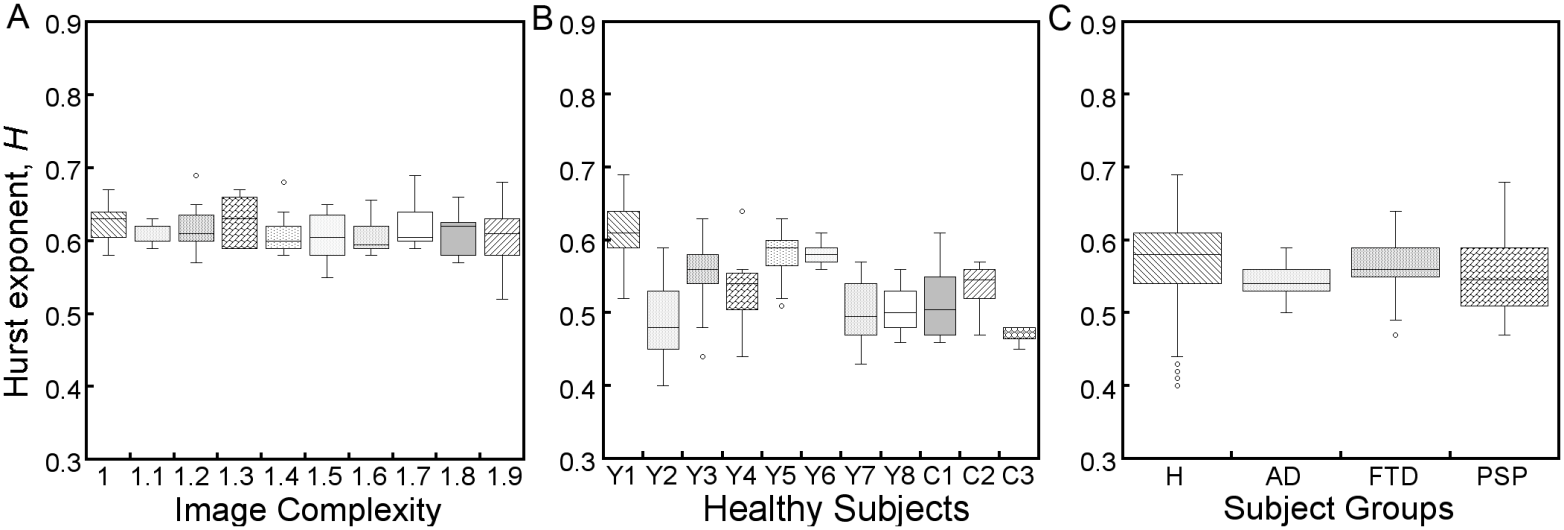
Box plots showing comparison of the Hurst exponent, *H*, across (A) image spatial complexity with the number of images analyzed n = 19, 5, 7, 6, 7, 8, 8, 6, 7 and 7 respectively, (B) individuals within the participant group H with n = 83, 21, 72, 8, 19, 9, 10, 6, 10, 18 and 3 respectively and (C) participant groups with n = 259, 24, 14, and 43 respectively.

To assess the effect that image complexity has on the gaze dynamics, we tracked the value of *H* for each of the participants across all the images they viewed. The resulting *H* values are shown for participant Y1 in Fig 4A. Box plots of *H* are grouped by image complexity. The accuracy of our analysis method was determined by generating several different artificial times series of known *H* and performing the variation method algorithm on them. The precision for determining *H* was found to be ± 0.03. To determine any significant differences between the image groups, a two-sample doubled sided t-test (*α*-level of 0.01) was performed with the Bonferroni correction for multiple comparisons. The Bonferroni correction adjusts the significance threshold to *α*/*m* where *m* is the number of groups being compared. In this case, the correction sets the significance threshold to 0.001. In all cases, there were no significant differences between *H* values across image complexity, with all instances giving *p*-values of at least 0.1 and the majority giving *p*-values larger than 0.5. The specific values for this participant (Y1) are listed in Table 1 as an example. Counter to our expectations, all participants showed no significant difference in the observed *H* value across image complexity indicating we see no change in the diffusive properties of gaze with image. These results suggest that the overall spatial complexity of an image does not affect the gaze dynamics during free viewing. Furthermore, scale invariance is seen in all cases of the Euclidean (non-fractal) images, even the simpler geometric images which have very low saliency. This suggests that scale invariant gaze dynamics prevail as long as participants are not actively fixating.

**Table 1:** *p*-values from double comparison two-sided t-test for image complexity groups for participant Y1. The value *C* represents the level of spatial complexity in the image, where *C* = 0 is the lowest and *C* = 0.9 is the highest. The significance threshold was determined by the Bonferroni comparison as 0.005.

	n = 19	n = 5	n = 7	n = 6	n = 7	n = 8	n = 8	n = 6	n = 7	n = 7
*C*	0	0.1	0.2	0.3	0.4	0.5	0.6	0.7	0.8	0.9
0	-	0.21	0.73	0.79	0.32	0.13	0.11	0.83	0.22	0.20
0.1	0.21	-	0.54	0.26	0.85	0.86	0.86	0.47	0.97	0.88
0.2	0.73	0.54	-	0.69	0.68	0.45	0.41	0.94	0.57	0.53
0.3	0.79	0.26	0.69	-	0.41	0.24	0.18	0.76	0.31	0.35
0.4	0.32	0.85	0.68	0.41	-	0.73	0.73	0.63	0.88	0.77
0.5	0.13	0.86	0.45	0.24	0.73	-	0.96	0.41	0.84	0.98
0.6	0.11	0.86	0.41	0.18	0.73	0.96	-	0.36	0.85	0.94
0.7	0.83	0.47	0.94	0.76	0.63	0.41	0.36	-	0.52	0.51
0.8	0.22	0.97	0.57	0.31	0.88	0.84	0.85	0.52	-	0.85
0.9	0.20	0.88	0.53	0.35	0.77	0.98	0.94	0.51	0.85	-

All of the participants’ *H* values were tested for normality using a two sample double-sided t-test (*α*-level of 0.05) for comparison with a normal distribution of the same mean and standard deviation (s.d.) of the data. All *p*-values resulting from normality testing are listed in Table 2 along with the mean value of *H*, *H*_*ave*_, and s.d. for each participant (*p*-values > 0.05 indicate no significant difference between the distributions and normality in the data). In the cases where the sample size was too small to reliably test for normality (n < 10), we also report the range of *H* values for the participant. Next specific Hurst exponents were compared across individuals within a given participant group. Fig 4B shows the box plots of the *H* values observed across all healthy participants (H).

**Table 2:** Hurst exponent, *H*, statistics listed for each participant. *H*_ave_ is the mean value per participant, s.d is the standard deviation and the *p*-values are those found from normality tests (*p* > 0.05 indicates normally distributed data). For small sample sizes (n <10), the range of *H* values for that participant is also reported.

Participant	n	*H*_ave_	s.d.	*H* range	*p*
Y1	83	0.62	0.03		0.87
Y2	21	0.49	0.06		0.79
Y3	72	0.56	0.03		0.95
Y4	8	0.53	0.06	0.48–0.56	0.68
Y5	19	0.58	0.03		0.56
Y6	9	0.58	0.02	0.56–0.61	0.90
Y7	10	0.50	0.04		0.63
Y8	6	0.50	0.03	0.46–0.56	0.40
C1	10	0.51	0.05		0.86
C2	18	0.54	0.03		0.56
C3	3	0.47	0.02	0.45–0.48	0.47
FTD1	2	0.62	0.03	0.60–0.64	0.92
FTD2	17	0.55	0.04		0.43
FTD3	6	0.58	0.02	0.56–0.60	0.64
FTD4	4	0.56	0.03	0.53–0.59	0.86
FTD5	14	0.52	0.04		0.84
AD1	16	0.54	0.02		0.55
AD2	5	0.56	0.03	0.51–0.59	0.47
AD3	3	0.48	0.04	0.45–0.52	0.22
PSP1	14	0.55	0.06		0.82

The observed Hurst exponents for individuals within a given participant group were compared using a two-sample doubled sided t-test with the Bonferroni correction for multiple comparisons. No significant differences were seen in the *H* values between individuals within the H group and individuals with degeneration in the frontal and anterior temporal lobes (FTD) and Alzheimer Disease (AD) also show no significant differences within each group. Lastly, *H* values were compared across all participant groups, including the participant with progressive supranuclear palsy (PSP), and reveal no significant difference. This is shown in the boxplot of Fig 4C. All *p*-values fall above the significance threshold determined from the Bonferroni correction for multiple comparisons (a significance threshold of 0.0125 in this case), see Table 3.

**Table 3:** a) Hurst exponent, *H*, statistics listed for each participant group. *H*_ave_ is the mean value per group, s.d is the standard deviation and the *p*-values are those found from normality tests (*p* > 0.05 indicates normally distributed data) on the groups data. b) *p*-values from double comparison two-sided t-test between participant groups. The significance threshold determined by the Bonferroni comparison is 0.0125.

a)	Group	*H*_ave_	s.d.	*p*	b)	Group	H	AD	FTD	PSP
	H	0.57	0.06	0.80		H	-	0.20	0.72	0.36
	AD	0.54	0.02	0.79		AD	0.20	-	0.20	0.22
	FTD	0.57	0.06	0.75		FTD	0.72	0.20	-	0.49
	PSP	0.55	0.06	0.41		PSP	0.36	0.22	0.49	-

Lastly, when we incorporate data from all participants in this study, we find that the gaze dynamics are quantified by a mean Hurst exponent of *H* = 0.57 (s.d. 0.06). This observed mid-range value suggests the visual system balances between a bias towards low and high frequency movements. Further, these results indicate that the statistics of gaze position for free viewing is consistent across all participants, even those with neural degeneration. This suggests that this behavioral feature may not be controlled for at the cognitive level of the visual system but is inherent to oculomotor processes occurring at an early stage of visual processing.

## Discussion

Our results indicate that gaze dynamics during free viewing are consistent with a random walk process where the movements come from a long-tailed Lévy distribution. This is in contrast with the dynamics of fixational eye movements observed when individuals were engaged in fixating on a given location in the visual field [10, 19]. Interestingly, during fixation a Hurst exponent of 0.35 was observed for movements on timescales larger than 20ms [10]. This value of *H* indicates antipersistent, or subdiffusive, movements. These types of movements are consistent with the demand of the task at hand- the need to keep the gaze localized on the intended fixation position. However, when an individual is engaged in the free viewing scenario of our study, *H* values indicate superdiffusive movements-the type necessary for visual exploration and that are suppressed during fixation. Within this context it appears that the visual system has different oculomotor strategies for different functional modes of viewing, which are uncovered by looking at the overall temporal structure of gaze dynamics.

Otero-Millan and colleagues have recently proposed a fixation-exploration continuum model in which eye movements have a common generator for exploration and fixation [20]. The persistence of scale invariant structure that we see in the gaze shift temporal dynamics at both ends of the spectrum from exploration to fixation is consistent with this model. Nevertheless, we see a change in the Hurst exponent with a change in task, from super-diffusive to sub-diffusive. An interesting question along these lines is whether the Hurst value varies continuously from exploration to fixation, as is seen with the saccadic measures [20], or whether it changes abruptly as the viewing mode is changed.

The H > 1/2 values revealed in our study indicate human gaze dynamics follow a strategy one would expect to be beneficial in exploration. Animals engaged in foraging exhibit super-diffusive behavior with *H* values which range from just above 0.5 to 1.24 [21]. Presumably, this range of values exists due to the wide range of conditions involved in the exploration in each case. The Hurst exponents we measure are at the lower end of superdiffusive behavior. Perhaps, just as is witnessed in animal foraging, the visual system takes on a range of *H* values depending on the particular viewing scenario (scene and task). In the case of the viewing paradigm of this study, the images used had relatively low visual content and the behavioral task was simply to look at the image and judge its aesthetic quality. The *H* value we observe could indicate that the visual system only engages weakly in exploration leading to a weak case of super-diffusion.

Mathematically, a higher value of *H* results in more rapid diffusion. It is surprising that increasing the fine scale spatial structure of the image had no effect the gaze dynamics. One might imagine a case where the resulting super-diffusive gaze dynamics could result from normal or Brownian diffusion interacting with a complex visual scene. However, our results show that the scaling is independent of the complexity of the visual scene. Images of high spatial complexity were included with the expectation that gaze dynamics would have a larger *H* value to effectively explore the more complex image. Even so, we find that image complexity does not induce changes to gaze dynamics.

To expand the ecological relevance of our study, we did a preliminary study in which 5 of our participants (C2, FTD1, FTD4, FTD5 and AD3) viewed photographs of natural landscapes. Example images are shown in Fig 1. Previous studies of human response to fractals employed two types of visual stimuli: computer simulations of fractals [17, 22–27] and photographs of natural landscapes [23, 24, 28–32]. So far in this study we have used the former and benefitted from the ability to adjust fractal complexity in a systematic, controlled fashion. We now incorporate the latter which display additional scale-invariant properties of natural scenery beyond the fractal shapes of the individual objects: these include the size distribution of the objects and the luminance contours established by the surface textures of the objects.

The measured gaze position data show scale invariant gaze dynamics in all cases consistent with the computer-generated fractal images, as shown in Fig 3E and 3F for participant FTD5. Furthermore, we observe no significant difference in mean Hurst exponent between the two sets of data, with a mean *H* = 0.54 (s.d. 0.06) for the natural landscapes.

It is also surprising that observed *H* values are robust across individuals. Individuals with Alzheimer Disease exhibit distinct differences in their visual processing, including impairments to visual cognition which impact visual memory and attention and affect visual search processes [33–35]. Patients with degeneration in the frontal and anterior temporal lobes (which includes the behavioral variant of FTD, semantic dementia and progressive nonfluent aphasia) and progressive supranuclear palsy often do not show impairment to higher level visual cognitive processes [36] while PSP patients show significant abnormalities in visually guided (reflexive) saccades and damage to the brainstem oculomotor system [37]. The fact that the AD, FTD and PSP participants all show no significant difference in gaze dynamics from the H participants indicates that this behavioral feature is fundamental to the human visual system and may not interact with higher level search processes or the control of reflexive eye movements. Universality suggests a common mechanism exists in the visual system which controls the generation of the shifts in gaze position and that this mechanism may be controlled at an early stage in visual processing.

How the human visual system responds to natural stimuli is a complicated question with multiple components at play. A scene and the observer can be either static or in motion, the scene itself can contain multiple levels of spatial complexity, and there are also a wide range of tasks during viewing. For this study, our goal was to simplify the natural viewing scenario to static black and white fractal images in which we controlled the spatial complexity of the natural pattern. Free viewing of static images while judging image appeal is only one of the many real world tasks the human visual system engages in and is just a first step in understanding the overall function of the visual system.

Future studies should apply times series analysis methods to gaze position data for participants in a variety of viewing scenarios such as active search to further determine the role task plays in gaze dynamics. In addition, analysis of gaze position data for participants in a variety of human developmental stages, in particular investigations of the gaze dynamics in infants, may tell us something about whether the gaze strategies develop or are robust to different stages of cognitive development.

Rucci and Victor recently addressed the functional importance that the temporal statistics of the class of fixational eye movements known as ocular drift have in image processing. They argue that these statistics play a significant role in the ability to see [13]. The dynamics of gaze position during visual exploration in free viewing of images dictates how the visual world is spatially represented over time to the neural system. We argue that the specific statistics of this strategy establishes a fundamental basis under which the visual system extracts information about the visual word. Efforts, such as ours, to understand what these statistics are and under what conditions they change can provide useful insight into how the human neural system acquires information about its environment.
